# Ploidy level and nucleotide variations in inflorescence dichotomous cultivars of plantain (*Musa* spp. AAB genome)

**DOI:** 10.1186/s12864-019-6083-5

**Published:** 2019-09-14

**Authors:** Ebiamadon Andi Brisibe, Norah Godwin Ekanem

**Affiliations:** 1 Department of Genetics and Biotechnology, University of Calabar, Calabar, Nigeria; 2 Bio-therapeutics/Bio-industrial and Plant Cell and Tissue Culture Research Laboratory, ACR Technologies Limited, 124 MCC Road, Calabar, Nigeria; 3 Department of Crop Science, Faculty of Agriculture, University of Uyo, Uyo, Nigeria

**Keywords:** Autochthonous genetic resources, Inflorescence dichotomous plantains, Genomic divergence, Phenological plasticity, Retrotransposons, Single nucleotide polymorphisms

## Abstract

**Background:**

Inflorescence dichotomy in *Musa* species is a rare developmental event which leads to the production of multiple bunches on a single pseudostem at fruiting. In spite of its fascinating attraction and seductive appeal, little is known about the cytogenetic basis and molecular mechanisms that could be ascribed to this phenomenon. To bridge this gap in information, an integrative approach using cytological fingerprinting and DNA ploidy level profiling (based on chromosome counting and flow cytometry) were assayed on five inflorescence dichotomous plantain varieties and a single-bunching cultivar that served as control. This was done to assess the number and behaviour of chromosomes on the one hand and single nucleotide polymorphisms identified during analysis of nucleotide variations on the other.

**Results:**

Chromosomes stained with aceto-orcein were very tiny, compact, metacentric and acrocentric, and differed both in number and ploidy level between the inflorescence dichotomous and single-bunching cultivars. The dichotomous plantains were mainly diploid (2n = 2x = 22) while the single-bunching ‘Agbagba’ cultivar was consistently a triploid (2n = 3x = 33), as revealed by histological chromosome counting and flow cytometry, implying that there was a high incidence of genomic divergence on account of ploidy variations among the different *Musa* cultivars. Molecular genotyping using single nucleotide polymorphisms detected on the *GTPase-protein binding* gene of the leaf tissue gene complex provided further evidence indicating that differences in the number of bunches among the inflorescence dichotomous cultivars could be ascribed to nucleotide diversity that was elicited by changes in amino acid sequences in the genome of the crops. Non-synonymous nucleotide substitutions resulted mainly from transversion (from purine to pyramidine and vice versa), tacitly implying that these changes were crucial and promoted a cascade of reactions in the genome that were, probably, responsible for the non-persistence of the dichotomization event(s) or the reversals in the bunch phenotype detected among the inflorescence dichotomous cultivars.

**Conclusions:**

This is the first report of cytogenetic fingerprints and nucleotide diversity detection among single- and multiple-bunching *Musa* cultivars. A clear distinction between the two groups was found that is indicative of variations both in ploidy level and nucleotide sequences. The pattern of single nucleotide polymorphisms provided profound clues suggesting that there was a high incidence of genomic divergence, due to random and unstable genetic events that were triggered by frequent spontaneous somatic mutations.

## Background

*Musa* is one of the three main genera of plants in the family Musaceae, which also includes the parthenocarpic edible plantains and bananas with diploid, triploid or tetraploid hybrids that have an extremely complicated origin involving natural hybridization, somatic mutation, and selection by early farmers for their tasty fruits [1]. Maintained by vegetative propagation, plantain (*Musa* species, AAB genomic group) is of extraordinary significance. It is amongst the few most important suppliers of dietary energy in the humid agro-ecological zones of the tropics [2], where it is cultivated and utilized as an essential food resource that is consumed by more than an estimated half a billion people, especially in Africa, the Caribbean Islands, Central Asia and Latin America. In fact, in many areas of sub-Saharan Africa, for example, plantain and a few other crops play an important socio-economic role as they constitute either a staple year-round calorie source or seasonal basic staple foods. The fruit is an important source of carbohydrates, proteins, minerals, β-carotene, ascorbic acid and moderate amounts of thiamine, riboflavin, nicotinic as well as folic acids [3, 4]. Plantain equally serves as a raw material in many rural-based cottage industries including the production of wine [5], vinegar and local beer, which is important nutritionally as it is described to be very rich in vitamin B due to the high yeast content in the brew. Aside from these, plantain is equally converted into high quality flour when the green, unripe fruit is peeled and the pulp dried and powdered. Such flour is known to be a very good source of energy and is usually preferred by diabetics as it has been demonstrated to be more digestible than that derived from any of the common cereals.

Up until recently, phenological studies have shown that plantain (*Musa* spp. AAB genome) flowers only once from an inflorescence that is composed of a single spike-bearing biseriate nodal cluster of flowers [6], which results in the production of a single bunch that would consist of several hands and fingers at fruiting. These may vary in size depending on several agronomic factors. Usually this process of flowering is the signal of an irreversible transition from vegetative to reproductive growth, and is accompanied by senescence of some of the leaves that completes the entire growth cycle of the plant. Lately, however, an enormous amount of naturally occurring genetic variations resulting in several forms of inflorescence developmental polymorphism which typically manifests by branching of the single spike with the stout peduncle during flowering has been observed, particularly in False Horn plantain cultivars [7, 8]. Phenotypically, cultivars with the inflorescence dichotomous traits do not differ from their single-bunching counterparts, and it is ordinarily not possible to distinguish between these two types of *Musa* varieties in the field during vegetative growth; prior to the development of the fruit bunch. Unlike the single-bunching cultivar that usually has a somatic chromosome number of 2n = 3x = 33 [2], the inflorescence of plantains showing developmental polymorphism is usually two, or sometimes even three, in number arising either from the same or completely different peduncles that results in the production of two or three bunches at fruiting [9, 10] on account of a single or, sometimes, double dichotomization events in the peduncle during floral development.

In recent years, these inflorescence dichotomous or multiple-bunching varieties of False Horn plantain have become quite commonly noticeable, especially in many parts of the oil rich south-eastern Niger Delta region of Nigeria, where expectedly the plants have become quite fascinating and appears to have a seductive appeal to local folks, primarily on account of the tremendous variations that exist in the morphology of the peduncles and the inflorescences borne on them. Bearing in mind that inflorescence dichotomous plantain varieties are suspected to be of great horticultural significance because of the inspiring possibility that they can provide additional benefits in terms of edible fruit weight arising from the two or three bunches produced on the single pseudostem [9], however, the cytogenetic basis and molecular mechanisms controlling the initiation and development of the different inflorescence polymorphic phenotypes have remained unclear due to paucity of scientific information. So far, only a few studies which considered the process of multiple bunch production to be associated with an advanced stage in the evolution of the crop [11] or that the phenomenon may be a stable genetic event, probably arising out of a spontaneous mutation from an already existing cultivar [9], have been reported.

At the moment, breeding efforts in *Musa* species including dessert bananas (AAA genome), cooking bananas (ABB genome) and plantains (with AAB genome) is encumbered by serious difficulties, especially due to the low levels of fertility and seed viability in many genotypes, structural heterozygosity, lack of characterized germplasm as potential parents for breeding [12], and the general absence of knowledge on the genetic factors responsible for important agricultural traits [13]. Given their enormous economic potentials, it is surprising that there have been no comprehensive studies of inflorescence and flower development in these *Musa* species. A thoroughly controlled scientific study that will define the nature of inflorescence development in the species can go a very long way in providing an insight into the cytological fingerprints and molecular mechanisms responsible for controlling the expression and development of inflorescence polymorphisms, which will obviously be crucial for understanding this phenomenon. Consequently the present study, which is part of a multi-dimensional investigation, was undertaken with two clear objectives in mind. First, it was our desire to unravel the cytological profile of these autochthonous plantain genetic resources through precise determination of their ploidy levels based on histological counting of metaphase chromosomes and flow cytometry. Our second objective was to characterize the fruiting inflorescence of the plant, whose development is initiated by intricate physiological stimuli that originate in the leaves, through a cascade of cytogenetic modulations mediated by members of the leaf tissue gene complex following detection of single nucleotide polymorphisms (SNPs) from the genomic sequences of the different inflorescence dichotomous cultivars studied in order to identify the candidate polymorphisms in the *GTPase–protein binding* gene in different plantain genomes that may be associated with known inflorescence dichotomous traits. This is essential as such details will not only increase our knowledge of the genomic diversity inherent in plantain but equally assist plant breeders and geneticists to understand the pattern and extent of the enormous gene pool of autochthonous genetic resources available within the *Musa* genus, which can be used for exploitation in future varietal improvement programmes of the crop.

## Results and discussion

### Morphological diversity of *Musa* inflorescence at fruiting

Prior to initiating cytological and molecular genotyping investigations, preliminary experiments were undertaken in parallel to examine the phenotypic diversity of bunches in the six different False Horn plantain cultivars at fruiting during the study period. The results showed that though an annual crop, plantain grows more or less as a perennial as the initial plant, usually referred to as the plant crop develops a corm and a pseudostem from which the original bunch is produced. After this initial bunch is harvested, the pseudostem dies and is being replaced by another from the stool that produces the first ratoon crop, which develops from a sucker originating from the corm. Similarly, a second ratoon crop is produced from the first ratoon and the cycle goes on repeatedly.

**Fig. 1:**
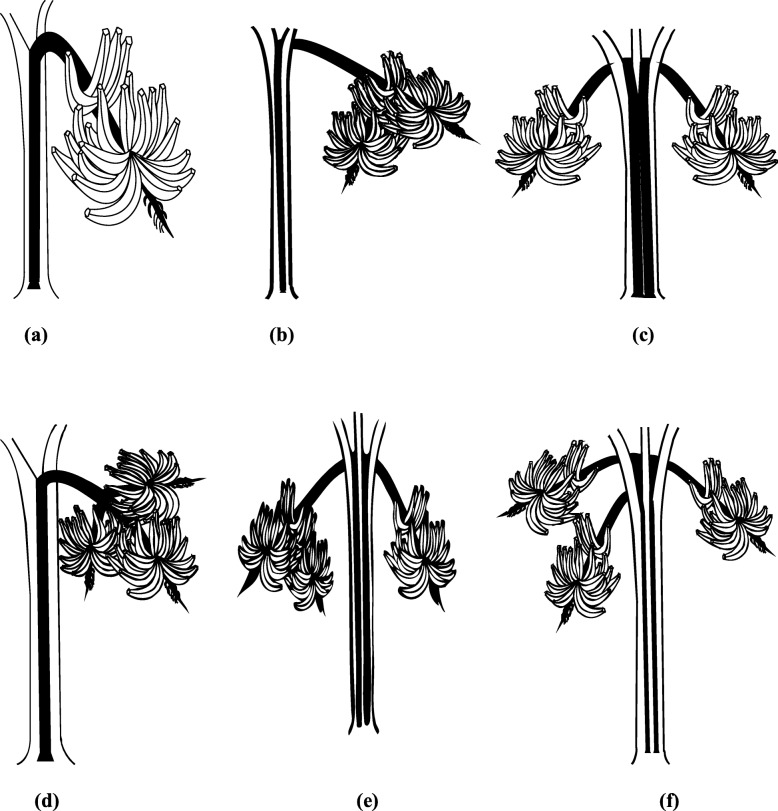
Morphological orientation of bunches in single-bunching and inflorescence dichotomous plantain cultivars: **a** A popular local False Horn plantain cultivar, which usually produces only a single bunch on one peduncle at fruiting that served as control in this study, **b** double bunches borne on one peduncle, **c** double bunches borne on two different peduncles, **d** triple bunches borne on one peduncle, **e** triple (three) bunches borne on two peduncles but with one bunch that is not fully expressed, and **f** triple bunches borne on three different peduncles

The fruiting pattern of the single-bunching ‘Agbagba’ cultivar during these crop cycles was consistent; always producing only a single bunch throughout the 5 years of the study. None of the 30 replicates of the cultivar during the plant, first or second ratoon crops showed altered bunch phenotypes as fruits developed normally with a concordance coefficient of 100% (Table 1). However, this was not the pattern with the double- and triple-bunching cultivars. These plants usually produced either 2 or 3 bunches on the same pseudostem in the plant crop, which could have resulted from either a single or double dichotomization events in the peduncle during floral development, as depicted in Fig. 1. Unlike plants of the single-bunching cultivar, there was no consistency in the number of inflorescences produced by any of the multiple-bunching cultivars during the first and second ratoon crops when reversals in bunch numbers were quite common and significant (*p* < 0.05) and resulted in low concordance coefficients (Table 1). For example, most plants of the double-bunching cultivars with bunches borne on one or two peduncles in the plant crop usually produced only a single bunch during the first ratoon and quite often, double bunches again in the second ratoon crop. Similarly, most plants amongst the cultivars with three bunches borne either on one, two or three peduncles in the plant crop equally produced either one or two bunches during the first ratoon, which subsequently reverted to single or double bunches during the second ratoon crop cycle, tacitly implying that inconsistency in inflorescence stability was found in greater abundance among the plants with a higher number of bunches; perhaps due to the absence of concurrence in genetic stability in the plants. This is not surprising as fruit formation in populations of predominantly inflorescence dichotomous plantain cultivars has been found to switch easily between multiple- and single-bunching phenotypic forms [10]. On account of the agreement between these two independent studies and the data provided in other related investigations [14], (Ubi GM, Brisibe EA: Molecular genotyping, nucleotide sequence variability and identification of phylogenetic relationships among inflorescence dichotomous cultivars of plantain (Musa spp. AAB genome), submitted), there is a growing body of evidence which strengthens the belief that reversals in dichotomous inflorescence in plantain provides an explanation of the inherent nature of the phenomenon and suggests that it may not be a genetically stable but rather random and an unstable genetic trait within the cultivars; a perspective that is quite at variance with the conclusion drawn earlier, that ascribed the dichotomization event in a ‘double bunching’ plantain cultivar as a consequence of stable genetic mutations arising probably from an existing plantain cultivar [9].

**Table 1:** Concordance coefficient analysis for nature of occurrence and persistence of inflorescence developmental polymorphism in plant, first and second ratoon crops in multiple-bunching plantain cultivars

Plantain cultivar	Plant crop	Ratoon^a^ crop	Concordance coefficient	Nature of occurrence	Persistence
‘Agbagba’	10	20 (20)	1.00	Normal	Persistent
DB1P	10	20 (13)	0.65	Random	Non-persistent
DB2P	10	20 (9)	0.45	Random	Non-persistent
TB1P	10	20 (3)	0.15	Random	Non-persistent
TB2P	10	20 (1)	0.05	Random	Non-persistent
TB3P	10	20 (0)	0.00	Random	Non-persistent

^a^Cumulative mean of number of plants during first and second ratoon crops

### Cytotype composition and flow cytometric profiling of single- and multiple-bunching plantain cultivars

**Fig. 2:**
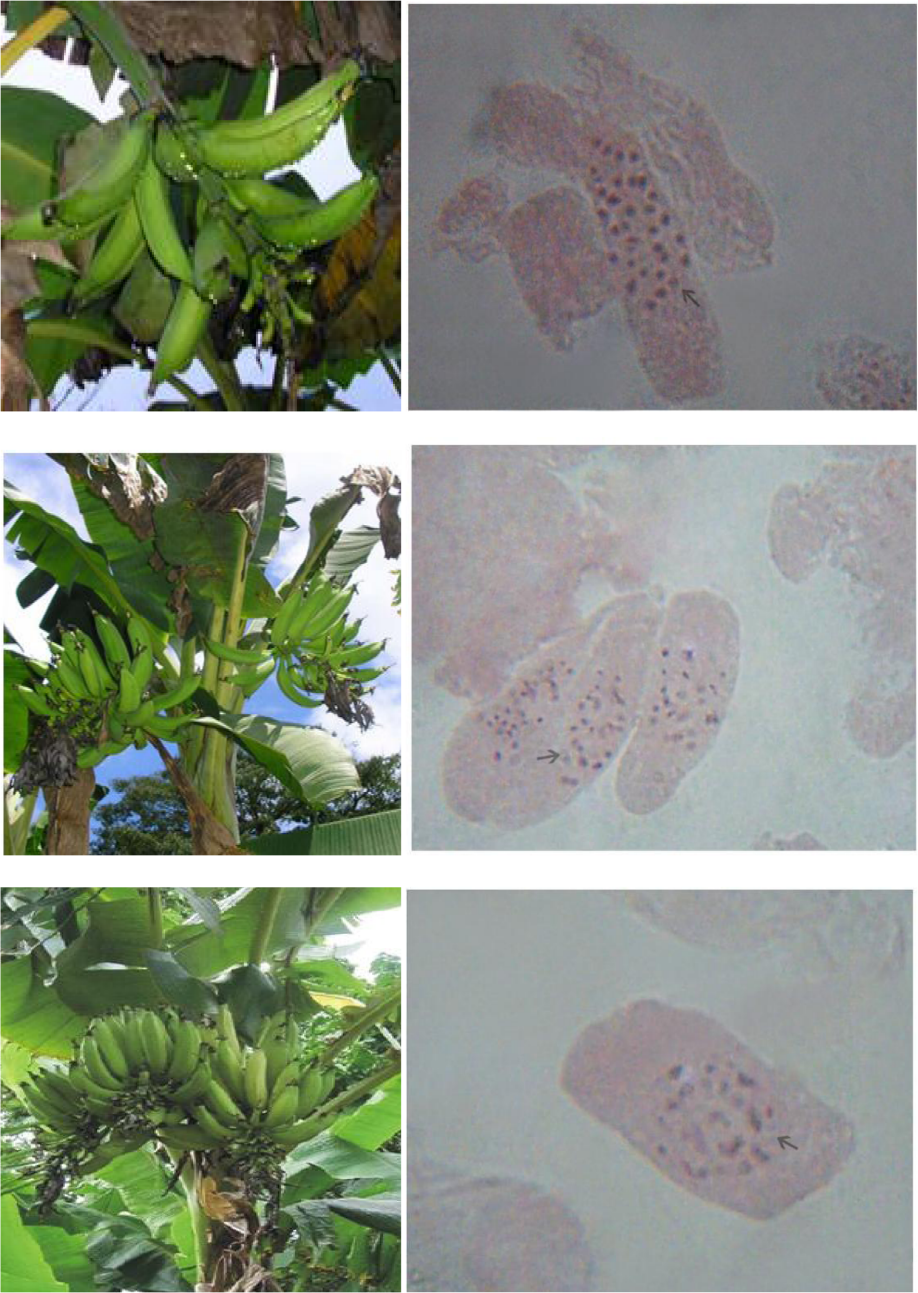
Different patterns of inflorescence orientation in plantain showing single, double and triple bunches on the left and the associated number of mitotic metaphase chromosomes counted in root tips of plants with the single bunch (2n = 3x = 33), double bunches (2n = 2x = 22) and triple bunches (2n = 2x = 22) on the right

Up until now, the ploidy level of multiple-bunching cultivars of *Musa* species has not been fully established, and this has drawn a significant degree of interest and was considered as a point of great importance in the design of the current study. To verify and confirm the intricate nature of the dichotomization events, the response from all six plantain varieties was therefore re-examined using root tip cells which were cytologically evaluated when the chromosomes were in the mitotic metaphase stage. Thereafter, the various levels of ploidy of the plants were equally examined by manually counting the chromosomes. Of great interest and significance was the observation that cells of the single-bunching ‘Agbagba’ cultivar did not exhibit any cytological differences from those of the inflorescence dichotomous plantains in terms of morphology. In fact, morphological characterization of the cells demonstrated that chromosomes in all six plantain cultivars were similar with no known chromosome-specific landmarks. They were all tiny and compact, with slight variations in size and could not be distinguished clearly by their length and centromere positions. In addition, root tip cells in all the cultivars equally revealed a spread of metacentric and acrocentric chromosomes with no telocentric structures though a few satellite-bearing chromosomes were observed (Fig. 2). Aside from these similarities between cells of the single- and multiple-bunching plantain cultivars, all other cytological features were uniquely and significantly different. For example, metaphase chromosomes in cells of the single-bunching plants, which served as control in the current study, showed the normal triploid mitotic chromosome number of 2n = 3x = 33 (as demonstrated in Fig. 2), in agreement with what has been reported previously by several research groups in French and Horn plantain varieties [15–19]. However, the reverse appears to be the case with chromosomes in all inflorescence dichotomous plantains, which were absolutely identical in morphology. Unlike the triploid mitotic chromosome number of the single-bunching ‘Agbagba’ varieties, most of the plants from the five inflorescence dichotomous plantain cultivars presented a diploid cytotype (2n = 2x = 22) following histological counting of chromosomes, irrespective of the number and orientation of the fruit bunches that they produced. Implicit in this observation is the fact that a majority of the multiple-bunching plantains evaluated in the current study were detected to have a diploid status (2n = 2x = 22), which is a position that is in agreement with other reports in some *Musa* species that were equally found to be diploid and considered to be genetically unstable [20–23].

This variation in chromosome number between the single and dichotomous bunching plantains may be due to a number of factors, which do not necessarily need to be mutually exclusive. First, it may be suggestive of a high incidence of genetic diversity within the *Musa* genus. Second, it may also be an indication that the species is still evolving and could be exploited as a tool for evolution and speciation studies [24]. Third, the chromosomal variations observed could as well suggest a genomic instability within these cultivars which can easily lead to mutation. Fourth, the deviation and drastic variation in chromosome number in the population of inflorescence dichotomous cultivars from the usually genetically stable, triploid cytotype (2n = 3x = 33) of the single-bunching plantain variety could also be explained on the basis of random and reversible insertional activation events that were caused by the presence of transposable genetic elements as earlier suggested [10]. Aside from these multiple explanations, the high level of similarity in morphology and number of chromosomes (2n = 2x) equally suggests that there could be a high degree of relatedness which is possibly an indication that the genomes of all inflorescence dichotomous cultivars may have coexisted within a single nucleus, perhaps on account of dominance of similar alleles responsible for the high degree of phenotypic interest displayed by them. Taken from the morphological point of view, it would be reasonable or even more appealing to treat all the multiple-bunching cultivars as genetically related, having diverged from the single-bunching cultivar several millions of years ago as revealed by the data in a related molecular phylogenetic and bioinformatics study (Ubi GM, Brisibe EA: Molecular genotyping, nucleotide sequence variability and identification of phylogenetic relationships among inflorescence dichotomous cultivars of plantain (Musa spp. AAB genome), submitted).

**Fig. 3:**
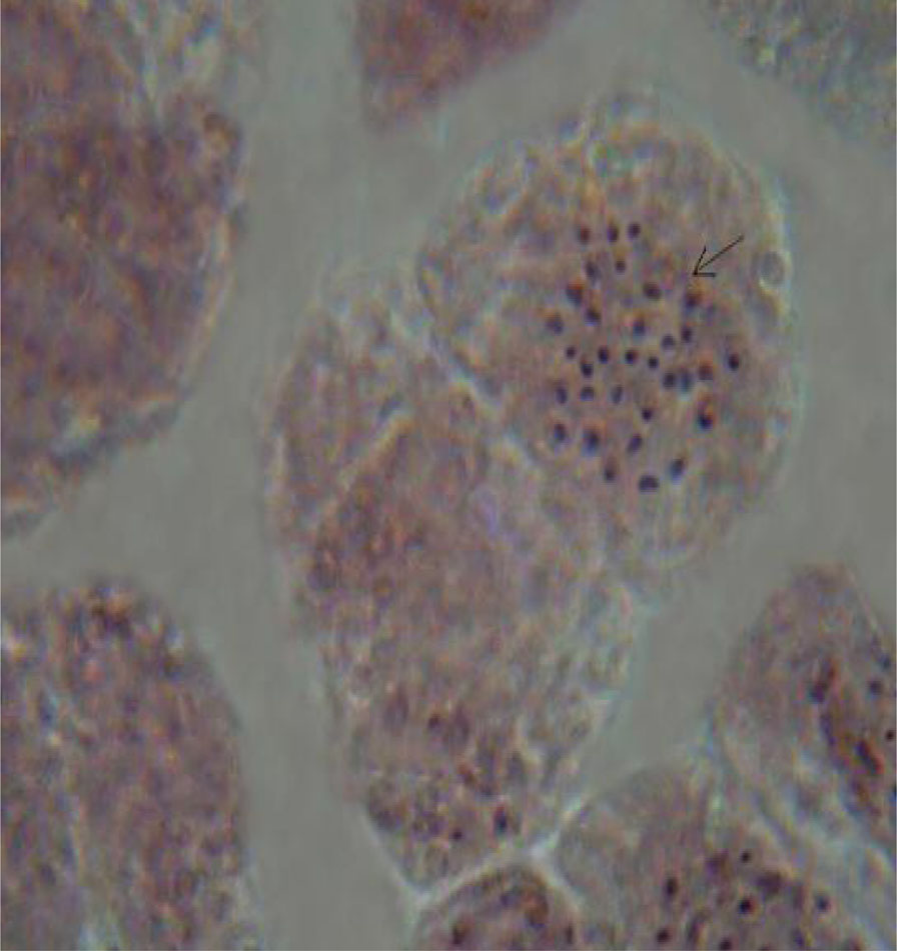
Mitotic metaphase chromosomes of root tops derived from a single-bunching cytotype (2n = 3x = 33) that was reversed from a triple-bunching cultivar in the first ratoon crop

**Fig. 4:**
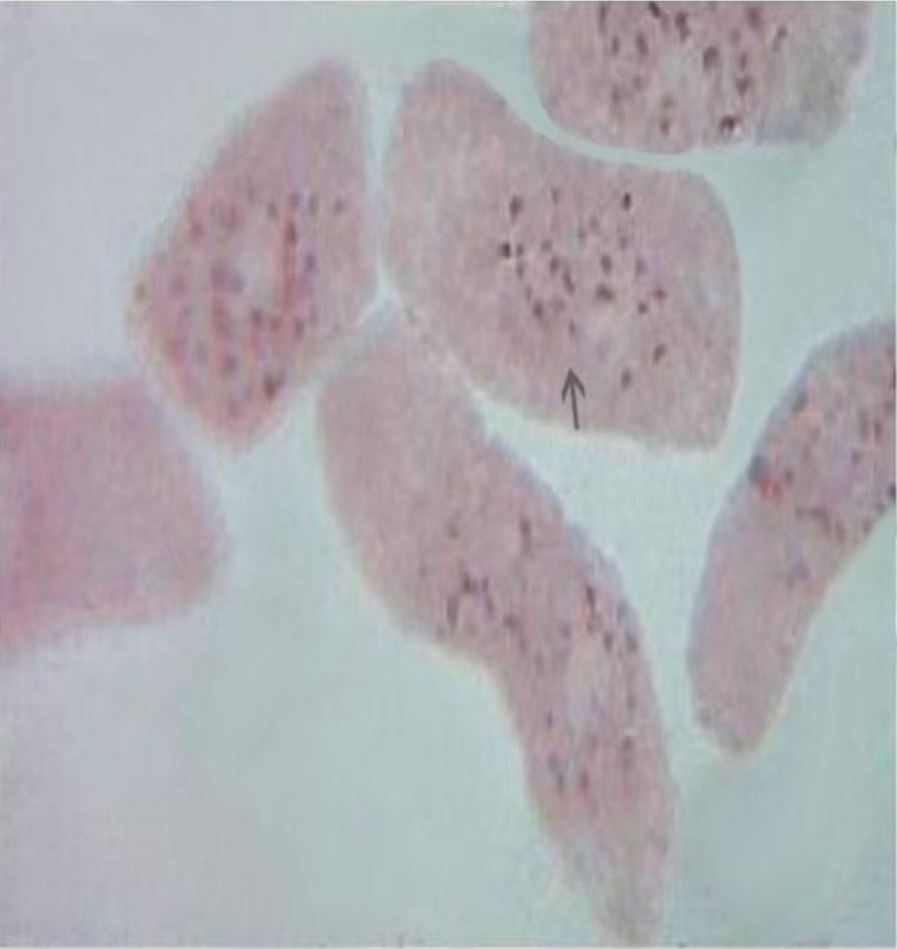
Mitotic metaphase chromosomes of root tops derived from a double-bunching cytotype (2n = 2x = 22) that was reversed from a triple-bunching cultivar in the second ratoon crop

One of the primary objectives of the current study was to determine if there were significant differences in ploidy level between the single- and multiple bunch-bearing plantain cultivars examined. Though the ploidy levels have been suggested above through manually counting the number of chromosomes from the microscopic slides (Figs. 2, 3 and 4), however, this still needed to be confirmed by flow cytometric profiling of cigar leaf tissues taken from the plant as well as the follower suckers derived from them in all ratoon crop cycles.

**Fig. 5:**
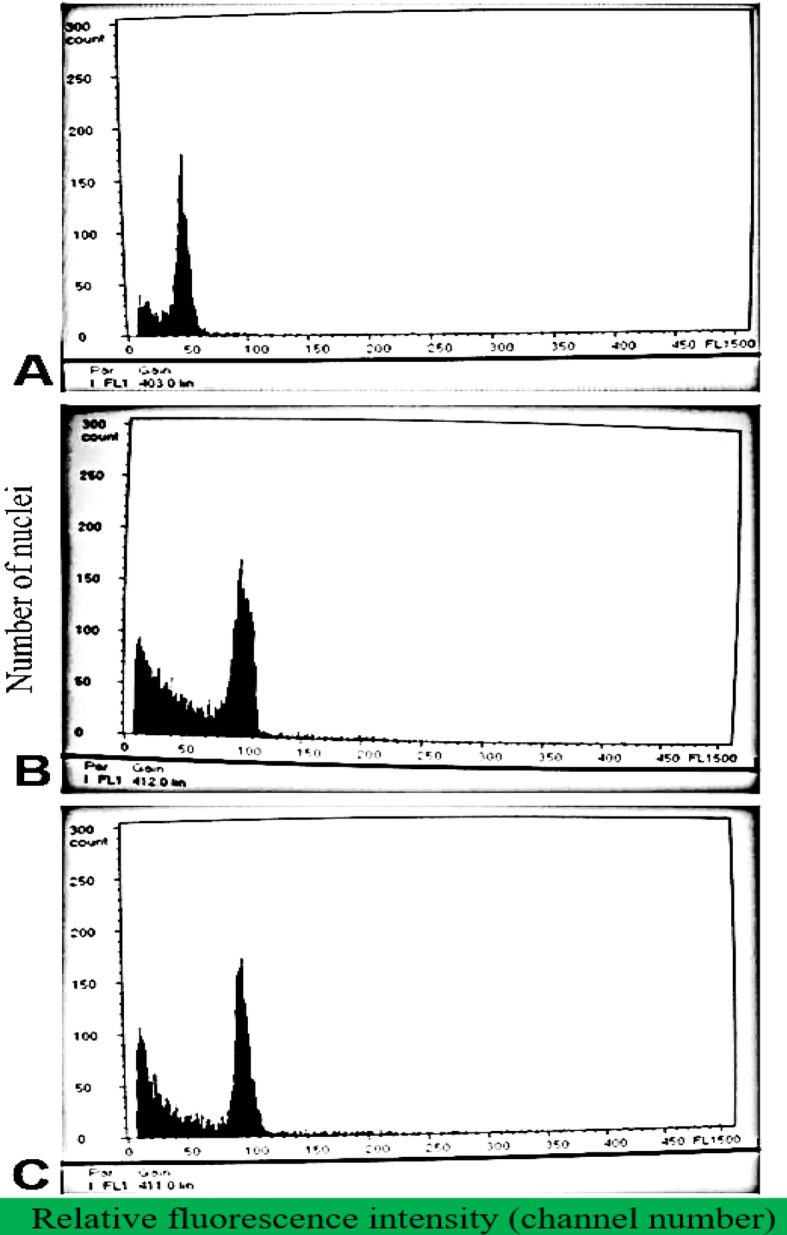
Histogram of relative nuclear DNA content of plantain cultivars showing different forms of inflorescence developmental polymorphism. The number of nuclei is shown on the vertical axis while relative DNA content, which is expressed as the fluorescence intensity (channel number) is shown on the horizontal axis: **a** Nuclear DNA content in leaves of a double-bunching plant in both the parent and subsequent generations (diploid, 2n = 50), **b** nuclear DNA content in leaves of a triple-bunching plantain cultivar in the parent generation (2n is peak centered around 100), **c** nuclear DNA content in leaves of a plant of the same triple-bunching cultivar during the second ratoon crop generation (2n = 100). Cigar leaves of plants of the single-bunching ‘Agbagba’ cultivar, which consistently presented triploid peaks were used as the internal control for measuring the DNA content of the inflorescence dichotomous cultivars

Considering the possible existence of ploidy polymorphisms among some of the multiple-bunching plants, the ploidy level of all six cultivars was thoroughly re-examined in a second series of experiments in the plant, first and second ratoon crops using DNA flow cytometric analysis to confirm the crop-cycle kinetics in the inflorescence dichotomous plants. Out of 100 plants (10 each for all 5 polymorphic variants during 2 crop cycles) evaluated in the first and second ratoon crops, 95 were diploid while only 5 exhibited tetraploid peaks (as demonstrated in Fig. 5 based on flow cytometry). On the basis of these results, it could be concluded that the greatest number or majority of the multiple-bunching plantains were diploids, which were genetically unstable resulting from mutations in some or all of the chromosomes due to instability of the ploidy level. Concomitant with these variations in ploidy status which were accompanied by differences in the chromosome number between the diploid variants amongst the inflorescence dichotomous varieties and the more stable triploid (2n =3x =33) that is usually considered to be the typical form of all plantain landraces (2), it could be speculated that the ploidy polymorphisms detected here may be reflections of the different genomic duplication events that may have taken place as the number of both bunches and crop cycles increased on account of speciation. Collectively, these observations tend to underpin the presence of a high incidence of genetic instability in the inflorescence dichotomous variants, thus highlighting the existence of a correlation between ploidy level and inflorescence orientation early in the crop cycle. However, this shifts favourably towards achieving the more stable triploid status in later generations, especially as the number of bunches produced on a single plant were reduced instead of increasing (Table 3). Given this scenario, it would definitely be of major horticultural significance for additional studies to be designed which would help to examine the precise basis of these ploidy shifts since cultivars with a higher number of fruit bunches appeared to be more unstable than those with the traditional single bunch (Tables 1 and 2).

**Table 2:** Ploidy description of inflorescence dichotomous accessions of *Musa* species analysed by chromosome counting and flow cytometry (in parentheses) during different crop cycles

Plantain cultivar	Ploidy level of plant crop	Ploidy level of first ratoon crop	Ploidy level of second ratoon crop
DB1P	2x (2x)	3x (2x)	2x (4x)
DB2P	2x (2x)	2x (2x)	3x (2x)
TB1P	2x (2x)	3x (2x)	2x (4x)
TB2P	2x (4x)	3x (2x)	2x (2x)
TB3P	2x (4x)	2x (4x)	3x (2x)

### Variation in nucleotide sequences of inflorescence dichotomous plantain cultivars

It is indicative from the data above that the differences in chromosome number and ploidy level among the different plantain cultivars evaluated provided some insights for the dichotomization events detected. However, the cascade of reactions in the genome which triggered these phenotypic polymorphisms still remains unclear. We reasoned that to have a better understanding of the molecular foundations underlying this highly unstable genetic trait, a possible strategy would be for nucleotide diversity studies and single nucleotide polymorphisms (SNPs) discovery in inflorescence dichotomous plantains to be initiated that would find diverged regions among members of a gene family including *GTPase–protein binding* gene in *Nicotiana tabacum*, *maturase K* gene in legumes, *PDV1* and *PDV2* genes in cereals, *rps II* gene in members of the Apocynaceae family, *rcbl* gene in cucurbits and *LRR-NBS* gene in legumes among many others, which are synonymous with the leaf tissue gene complex in *Musa* species. The genomic DNA extracted from the six different plantain cultivars were amplified clearly and distinctively at the specific base pairs with the *GTPase –protein binding* gene when compared to primers designed from the other gene complexes.

**Fig. 6:**
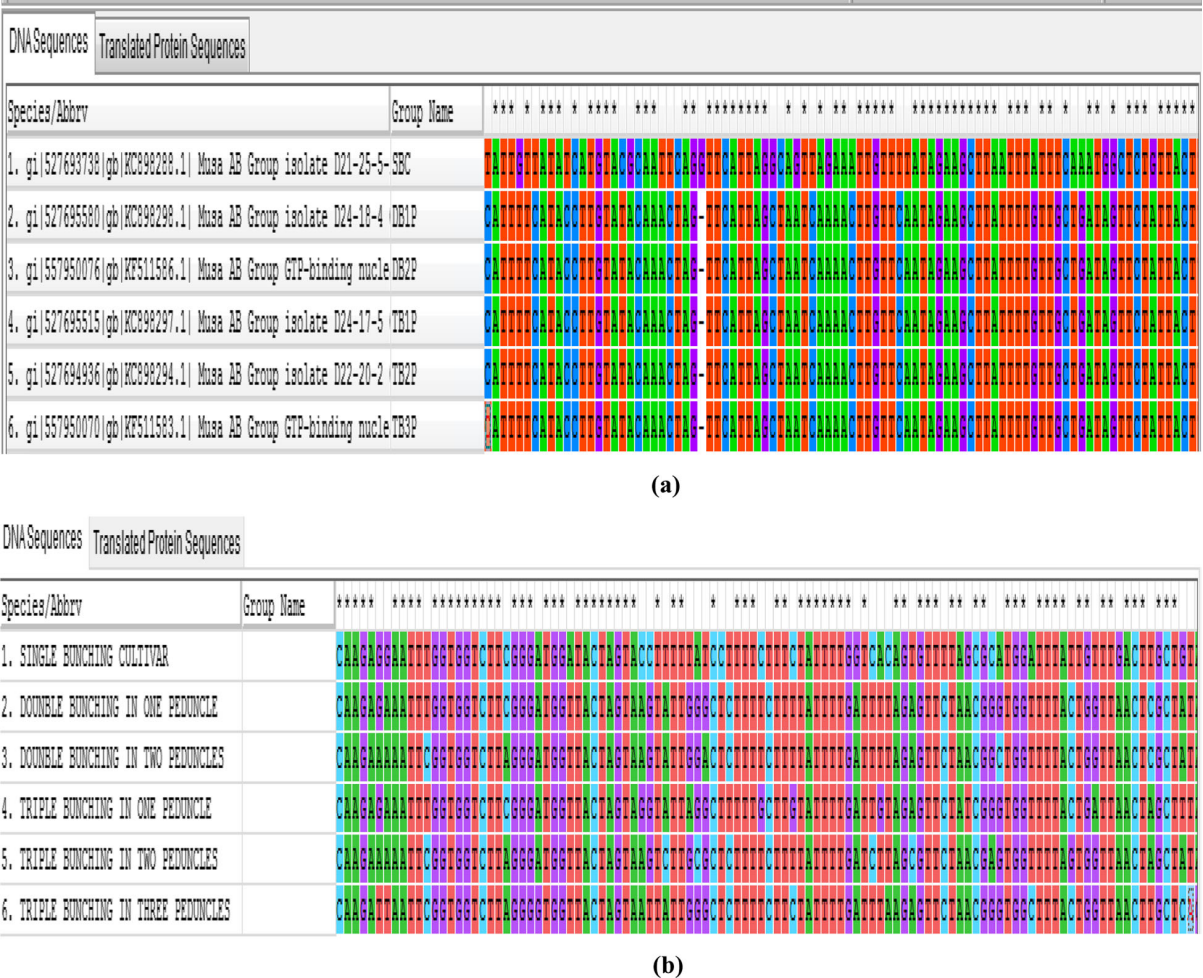
Single nucleotide polymorphism analysis in several loci using multiple sequence alignments for a single-bunching (SBC) and 5 inflorescence polymorphic (DB1P to TB3P) plantain cultivars showing positions of (**a**) nucleotide deletion and (**b**) nucleotide swaps

Multiple sequence alignments created from pair-wise alignments used to select the highly conserved variation-enriched regions of the *GTPase-protein binding* gene are presented in Fig. 6. This identified two things. First, there was a major nucleotide deletion in the inflorescence dichotomous cultivars (Fig. 6a). Second, there was a high level of nucleotide diversity in the genome of the inflorescence dichotomous cultivars, which are represented as nucleotide swaps in Fig. 6b, that may have resulted from duplicated loci or allele separation from the ancestors at the time of divergence from the single-bunching cultivar.

The key descriptive details for the single nucleotide polymorphic loci, types of nucleotide substitutions and amino acid changes in the genomic sequences of the different cultivars are presented in Tables 3 and 4, respectively. As expected, the single-bunching plantain cultivar was genetically stable; it possessed a higher number of amino acids with the least number of nucleotide changes or substitutions, perhaps on account of the presence of nucleotides in the exon or conserved region of the gene (Ubi GM, Brisibe EA: Molecular genotyping, nucleotide sequence variability and identification of phylogenetic relationships among inflorescence dichotomous cultivars of plantain (Musa spp. AAB genome), submitted). Invariably, all the base pair variations in its genome involved only a change in position of the nucleotides such as from guanine to adenine and adenine to guanine, without the prediction of any concomitant changes in the amino acids (Table 3).

**Table 3:** Single nucleotide polymorphisms (SNPs), nucleotide substitutions, and amino acid changes in *GTPase-protein binding* gene of the leaf tissue gene complex of single-bunching and inflorescence polymorphic plantain cultivars

Plantain cultivar	Number of SNP sites	Nucleotide substitution	Total number of nucleotide changes	Amino acid changes
Agbagba’	55	Pu – Pu	26G > A / 12A > G	Lys 14 Lys
DB1P	82	Py – Pu	46 T > G / 23C > A	Gly 27 Val
DB2P	77	Pu – Py	33A > C / 12G > T	Leu 16 Pro
TB1P	81	Py – Pu	40 T > A / 18C > G	Val 22 Lys
TB2P	79	Py – Pu	33A > T / 19C > A	Leu 23 Gly
TB3P	86	Pu – Py	28G > C / 21 T > G	Gly 19 Val

*SNP* Single nucleotide polymorphism

**Table 4:** Types of nucleotide substitutions showing synonymous and non-synonymous mutations in *GTPase-protein binding* gene of the leaf tissue gene complex of single-bunching and inflorescence polymorphic plantain cultivars

Plantain cultivar	Number of SNP sites	Nucleotide substitution	Type of nucleotide substitution	Type of mutation
‘Agbagba’	55	Pu - Pu	Transition	Synonymous
DB1P	82	Py – Pu	Transversion	Non-synonymous
DB2P	77	Pu – Py	Transversion	Non-synonymous
TB1P	81	Py – Pu	Transversion	Non-synonymous
TB2P	79	Py – Pu	Transversion	Non-synonymous
TB3P	86	Pu – Py	Transversion	Non-synonymous

*SNP* Single nucleotide polymorphism

Now a particularly intriguing and significant aspect of our observations was the revelation that transitional and synonymous point mutations in single nucleotide polymorphisms (that involved a change from purine to purine and pyrimidine to pyrimidine or leucine to leucine and lysine to lysine) were only peculiar to the single-bunching plantain cultivar. This is obviously an indication of a silent mutation with no changes in the amino acid sequence, which consequently does not lead to an alteration in the expression and function of the gene. This provides a cue of the stimulating possibility that the absence of any changes in the amino acid sequence may have accounted for the genetic stability that was detected in all replicates of the single-bunching plantain cultivar. In contrast to this, a number of transversions and non-synonymous single nucleotide changes were detected in the coding region of the gene in the phenotypic variants (as presented in Table 4), which implies that there were amino acid substitutions that resulted in alternative receptor isoforms in both the double- and triple-bunching plantain cultivars. These changes can be speculated to have contributed to the phenotypic plasticity that was detected in the inflorescence dichotomous cultivars. Unlike the single-bunching cultivar, the fundamental nucleotide changes observed in the inflorescence polymorphic phenotypes were intriguingly from purine to pyramidine and pyramidine to purine while some of the typical amino acid changes in the different cultivars included glycine to valine in DB1P, leucine to proline in DB2P, valine to lysine in TB1P, leucine to glycine in TB2P, and glycine to valine in TB3P, respectively. Such diverse manifestations resulting in nucleotide substitutions and changes in the types and positions of amino acids equally suggest highly complex molecular cascades that can be associated with alterations in both gene expression and function in subsequent cropping cycles. It is speculative from these details, therefore, that the high mutation rates characteristic of both nucleotide base pairs (Fig. 6a and b) and amino acid sequences could have led to the high genetic instability detected, which may have developed in the inflorescence dichotomous cultivars as a consequence of incomplete penetrance or pleitropy. It is equally possible that the genomic changes detected could be strongly associated with other factors including epigenetic modifications, which represent an important source of natural variation, that are potentially reversible and provide a flexible mechanism for the plants to respond to harsh genomic changes, as frequently observed at the early stages of polyploidy formation that could lead to the development of different epigenetic variants.

There are no misgivings that the genomic consequences detected above, especially the changes in nucleotide and amino acid composition of the *GTPase protein binding* gene in the inflorescence polymorphic variants may be fascinating. Unfortunately, there are no previous reports on how these variations could affect the phenotypic expression of plantain inflorescence until now. All the data generated in the current study, therefore, should be considered as preliminary in so far as whether or not the SNPs contribute to individual variability in inflorescence orientation remains an open question. Invariably there are still important lessons to be drawn here as the factors which may have triggered the molecular cascades that engineered the different ploidy levels or genomic nucleotide variations responsible for the dichotomization events in the multiple-bunching plantain cultivars have not been ascertained. At this moment, we can only speculate that DNA methylation may be partly or entirely responsible for the epigenetic signalling that resulted in the formation of the inflorescence dichotomous agronomic trait. Like with most plant species which are exposed to heterogeneity in the environment, where new abiotic and biotic stress factors including rapid changes in climate, pollution, pest invasiveness and diseases are introduced, the inter- and intra-specific inflorescence polymorphisms observed may be reflections arising from stress factors in the environment. Consequently, the impressive phenotypic plasticity observed among the multiple-bunching plantains could be considered as one of the major adaptive means by which the plants may have tried to cope with environmental factor variability through exhibition of remarkably diverse and plastic developmental complexities [25]. The attraction in this point of view, however hypothetical the causative agents may be, lies in the fact that it focuses attention upon two intriguing developments which are not mutually exclusive. To begin with, it has been highlighted that under rapid climate change or adverse environmental or cell-context signaling, phenotypic plasticity rather than genetic diversity is likely to play a crucial role in allowing plants to persist in their usual environments. Given this scenario, it is assumed, therefore, that the plethora of inflorescence developmental polymorphisms detected in the plantains may have arisen as a result of selective pressure in the environment, most probably due to air, soil, and water contaminations arising from pollution that is occasioned by the rapidly increasing incidences of oil spills from oil exploration and exploitation activities in the Niger Delta region [26], where uncontrolled gas flares containing invincible and noxious gases, especially nitrogen oxide and sulphur dioxide, react with tiny droplets of atmospheric water and vapours to form acid mists, which have been reported to prompt changes in plants and animals [27]. Aside from this, there is also evidence currently emerging which have actually provided strong existential data linking these high levels of acid mists or ‘acid rains’ to increased and extensive damage to the biota and health of the people in the area through an escalating number of malignant melanomas and environmental pollution that is manifested in increasing destruction to water, soil, animals, forests, and fishery resources [28–31] and retardation in the growth of plants through stimulation of damages [32] during budding, flowering and photosynthesis.

## Conclusions

The present study represents the first attempt at using an integrative approach involving cytological and nuclear DNA ploidy profiling and molecular fingerprinting data to address the underlying genetics of inflorescence polymorphism in plantain, a phenomenon that has lately become noticeable in False Horn plantain cultivars. The results presented here show that while the five inflorescence dichotomous cultivars consisted mainly of diploids (and an highly inconsequential number of tetraploids) with several polymorphic variants that were genetically unstable, the single bunch-bearing ‘Agbagba’ cultivar was triploid and genetically stable. These findings, therefore, tend to indicate that the dichotomization events leading to inflorescence developmental polymorphism in plantain are likely caused by spontaneous mutations, which are unstable and grossly affect both the mitotic chromosome number as well as ploidy level of the plants. Additionally, the single nucleotide polymorphism findings also tend to suggest that it was some small changes in the nucleotide sequences that were the primary trigger that orchestrated the molecular cascades that were responsible for the phenotypic plasticity detected in the inflorescence architecture of the plants. In association with ploidy level, the SNPs found in a fragment of the *GTPase–protein binding* gene of the leaf tissue gene complex have provided a clearer interpretation of the phenomenon of inflorescence dichotomy in *Musa* species, perhaps, on account of rapid changes in climate or pollutions in the environment.

Bearing this is mind, molecular markers such as retrotransposons and simple sequence repeats (SSR) or microsatellites, which are more informative for the discrimination of closely related genotypes and are considered to be of great value in fingerprinting, mapping, and genetic analysis of plants can be used for discriminating between and within the two groups of plantains evaluated in this study. Thus far, retrotransposon- and microsatellite-based molecular markers have been readily utilized for purposes of genetic analysis, including an estimation of the genetic diversity of plants [33], determination of the relationships between plant accessions, elucidation of evolutionary relationships, as an aid in the taxonomic classification of many plants including tropical pasture grasses [34–37] as well as in the molecular genotyping of important crops such as rice [38], orphan legume species [39, 40] and banana and plantain [12]. Consequently, their use in the evaluation of dichotomous bunching plantain cultivars will be in order and highly recommended as this will help to pinpoint the degree of divergence within them on the one hand and between them and the single bunching cultivar of False Horn plantains on the other. In addition, utilizing molecular markers which can help to identify many genetic polymorphisms would equally make it feasible to address the relationship, if any, between morphological and genomic variations amongst multiple (dichotomous) bunching plantain varieties that consequently will provide substantial insights in directing introgression and molecular breeding strategies in these varieties of plantain in the future.

## Methods

### Plant material

Plant material used in this study was derived from six different *Musa* cultivars, which are depicted and described in Fig. 1. Ten replicates each of all six cultivars with the different bunch orientations were initially collected as sword suckers from different fields of small-holder farmers spread across south-eastern Nigeria, and maintained as field plants at the Teaching and Research Farm, University of Uyo, Uyo, Nigeria (5° 03′N, 7° 56′ E, and 65 m.a.s.l.). The farm lies in an area within the rainforest zone with a mean relative humidity of 86%, annual rainfall of 2000 - 2500 mm and minimum and maximum daily temperatures of 23 °C and 34 °C, respectively.

The plant accessions were identified by Prof. Greg Akpan, Department of Botany, University of Uyo, Uyo, Nigeria, while voucher specimens have been deposited in the Botanical Garden, University of Calabar, Calabar, Nigeria.

In the current study, *Musa* cultivars were examined in the field over a period of three crop production cycles between 2010 and 2015. During each of these crop cycles (that is, plant, first and second ratoon crop generations), the phenotypic orientation of the fruit bunches of individual plants was recorded at regular intervals from floral initiation to fruit maturity and grouped according to the number of bunches borne on the pseudostem.

Concordance correlation coefficient, which is used to evaluate the agreement between paired readings, was undertaken to determine the proportion of plants that showed the same inflorescence phenotype in the three production cycles, excluding dead plants. The concordance coefficient, denoted CC, was calculated as previously recorded [10]:

$$ \mathrm{CC}=100\ \mathrm{x}\frac{\sum \mathrm{iNii}}{\sum \mathrm{iNii}+\sum \mathrm{ijNij}},\mathrm{i}\ne \mathrm{j} $$

where Nii is the number of plants that expressed the same number of bunches in the plant crop and in the ratoon crop, Nij is the number of plants that produced different numbers of bunches in the plant crop and in the ratoon crop, and the subscripts i and j refer to the number of classes in the plant crop and the ratoon crop, respectively. A concordance coefficient approaching zero would indicate completely random occurrence of the different inflorescence classes across crop cycles. In contrast, a coefficient near unity would suggest a high probability that a plant expressing a given phenotype in a given crop cycle would express the same phenotype in subsequent crop cycles.

### Cytological evaluation and chromosome counting

For cytological evaluation, actively growing root tips approximately 1.5–2.0 cm in length were harvested early in the morning from each plant when the orientation of the inflorescences on the peduncle became clearly discernible. The root tips were rinsed in running tap water to remove all soil particles and debris. Subsequently, secondary roots about 10–25 mm long and 1–2.5 mm thick were carefully harvested using a pair of forceps and immediately pretreated in 2 mM aqueous solution of 8-hydroxyquinoline for 2–4 h.

To highlight the structure of the chromosomes and assess the potential cytological impact, pretreated root samples were fixed in an ice-cold 3:1 binary mixture of ethanol and acetic acid [41] to induce cell bursting and kept for 12–24 h before use. All samples were thereafter hydrolysed in 9% HCl for 5–10 min, preserved in 70% ethanol solution and stored at 0 - 4 °C until required for squashing after fixation.

The white denser portion of each root tip (about 2 mm in length) was excised and squashed in a drop of FLP orcein stain [41] under a cover glass. For chromosome counting, cells were flattened out by pressing firmly with a thumb and thereafter stained with aceto-orcein. Slides were prepared and observed under a binocular microscope (Olympus Corporation, Tokyo, Japan). Images of mitotic chromosomes at the best metaphase views were captured using a micro-digital camera (Canon 960, Canon Corporation, Japan) at a magnification of 1000x. Chromosomes were subsequently counted, measured manually from camera Lucida drawings and processed using image analysis software (Lucia, version 4.21). For every plantain variety, at least ten metaphase cells showing well scattered and contracted chromosomes were counted.

### Estimation of DNA ploidy level

To confirm the reliability of the conventional chromosome counts, results of ploidy estimates were supplemented by flow cytometry, as described previously with minor modifications [42, 43]. Briefly, 50 mg of cigar leaf samples taken from the six different plantain cultivars were chopped with a razor blade in 0.5 ml of 0.5 × WPB buffer [44] and 1 ml of the same buffer was added. The homogenate was filtered through a 50 μM nylon mesh and incubated with 50 μg ml–1 of RNase (Sigma-Aldrich) and 100 μg ml–1 of propidium iodide for 10 min. The resulting samples were analyzed with a ploidy analyzer (Partec PA) to estimate the cytotype [42] using cigar leaves of plants of the single-bunching ‘Agbagba’ cultivar, which consistently presented triploid peaks as the internal control for measuring the DNA content of all the inflorescence dichotomous cultivars.

### Genomic DNA extraction and sequencing of *Musa* cultivars

Genomic DNA was extracted from fresh cigar (unopened) leaf tissue of suckers of each cultivar using the BioSprint 96 DNA Plant Kit protocol and materials (Qiagen, Venlo, The Netherlands). DNA sequencing involved using 2 μl of Big Dye terminator (v 3.1) cycle sequencer. A 4 μl 5X sequencer buffer was prepared using 1 μl 3′ primer (3.3 picomol) which was about 4X lower than the concentration of the normal PCR in 10 μl of distilled water and 3 μl of purified DNA sample to give a total of 20 μl which was vortex to mix properly. PCR programme was run for 35 cycles. The sequencer samples were prepared by mixing 20 μl of the DNA sample extract, 50 μl of absolute ethanol, 2 μl of 3 M sodium acetate, 2 μl of 125 mM EDTA and incubated at room temperature for 15 min. The entire mixture was centrifuged at 15 rpm at 20 °C for 20 min before discarding the supernatant. A 70 μl aliquot of 75% ethanol was added, shaken in a vortex machine and centrifuged for 15,000 rpm at 20 °C for 5 min. Thereafter, the supernatant was discarded, dried in desiccators and re-suspended in a buffer for the sequencer. A homology-based PCR method in which a dinucleotide primer: Forward (TTTTAACCAAGGTTTTGGGGAA) (TG) and reverse (AAAATTGGTTCCAAAACCCCTT) was used to amplify the genomic DNA before sequencing of a subgroup of leaf tissue genes complex, which encodes regulatory proteins conserved throughout a wide variety of plants. In the DNA sequencer, capillary electrophoresis for size separation, detection and recording of dye fluorescence with data output was carried out as fluorescent peak trace chromatograms. Nucleotide sequences were edited using Staden’s [45] Package.

### Single nucleotide polymorphism genotyping of *Musa* cultivars

In *Musa* species, especially in plantains and bananas, the inflorescence develops as a terminal bud from the true stem or corm underground which grows through the centre of the foliage leaves that form the pseudostem, emerging at the top in the centre of the leaf cluster. This development is mediated through a cascade of cytogenetic modulations that are similar to those encoded by multiple genes in cereals. Consequently, primers were designed and tested against different combinations of proposed markers for control of epigenetic gene expression including *GTPase – protein binding* gene in *Nicotiana tabacum, Maturase k* gene in legumes, *PDV1* and *PDV2* genes in cereals, *rps* II gene in members of the *Apocynaceae* family, *rcbl* gene in cucurbits, and *LRR-NBS* gene in legumes, among many other genes which are synonymous with the leaf tissue genes complex in *Musa* species.

Nucleotides of the *GTPase–protein binding* gene which has an epigenetic control of gene expression in *Nicotiana tobacum* were aligned in the current study using the molecular evolutionary genetic analysis (MEGA 7.0) software. Pairwise sequence alignments were used to select the highly conserved regions, which were used to create a multiple sequence alignment (MAS) FASTA file to detect the presence of nucleotide substitutions and changes in the sequences amplified, sequenced and analyzed within the single and multiple bunching plantain cultivars evaluated to identify SNPs present in each of the cultivars.

The number of single nucleotide polymorphisms (SNPs) and the changes in nucleotides in the SNPs were recorded as displayed by the software results. These were used as basis to identify nucleotide diversity and discover single nucleotide polymorphisms (SNPs) in all five inflorescence dichotomous plantains that were compared with those of the single-bunching ‘Agbagba’ cultivar.

### Statistical analysis

Phenotypic data on the different parameters collected were subjected to analysis of variance (ANOVA) using Statistical Analysis Systems (SAS) software according to the procedure outlined for randomised complete block design (SAS 9.2 version 2009). Fisher’s Least Significant Difference (LSD) was used to separate means at 5% significant level (*p* < 0.05).

## Data Availability

All data generated or analysed during this study are included in this published article. However, *Musa* samples exhibiting inflorescence developmental polymorphism are available with authors.

## Abbreviations

(ii) DB2P: Plants with double bunches borne on two separate peduncles
(iii) TB1P: Plants with triple bunches borne on a single peduncle
(iv) TB2P: Plants with triple bunches borne on two peduncles
(v) TB3P: Plants with triple bunches borne on three different peduncles
DB1P: Plants with double bunches borne on a single peduncle
SBC: Single bunching plantain cultivar (‘Agbagba’) that served as control in the current study
